# Evidence from partially valid cueing that words are processed serially

**DOI:** 10.3758/s13423-022-02230-w

**Published:** 2022-12-22

**Authors:** Miranda Johnson, John Palmer, Cathleen M. Moore, Geoffrey M. Boynton

**Affiliations:** 1 Department of Psychology, University of Washington, Seattle, WA USA; 2 Department of Psychological and Brain Sciences, University of Iowa, Iowa City, IA USA

**Keywords:** Attention, Selective attention, Spatial cueing, Visual word recognition

## Abstract

There has been a longstanding debate about whether lexical and semantic processing of words is serial or parallel. We addressed this debate using partially valid cueing, where one of two words is cued. The cue was valid on 80% and invalid on the other 20% of the trials. The task was semantic categorization, and performance was measured by accuracy. The new feature was to limit attentional switching using a postmask of consonants that closely followed the presentation of words. We found a large effect of cueing and, most importantly, performance for the uncued word was at chance. This chance performance was consistent with serial processing, but not with typical parallel processing. This result adds to the evidence from other recent studies that the lexical and semantic processing of words is serial.

## Introduction

In reading, the eyes move to fixate groups of words that can be processed in or near the fovea. There is little doubt that such groups of words are processed serially from fixation to fixation (for a review, see Yeatman & White, 2021). In contrast, there is a longstanding debate over whether individual words within a fixation are processed serially (e.g., Engbert et al., 2005; Reichle et al., 1998). In this article, we address this debate using word recognition/categorization for one versus two words.

Previously, the question of serial versus parallel processing of words has been addressed by a variety of paradigms, which are reviewed in the *General discussion*. This article was inspired by the recent studies of White et al., (2018, 2020). Participants made semantic judgments about words that appeared on either side of fixation. In a single-task condition, participants were cued to a single spatial location with a target word. This was compared to a dual-task condition in which participants were cued to two spatial locations with target words. Importantly, White and colleagues used a postmask to minimize attentional switching between words. They found that divided attention effects for their semantic judgment task were consistent with an all-or-none serial model in which only one word can be processed on a trial. Additionally, a correct response for one side in the dual-task condition was associated with an incorrect response on the other side (White et al., 2018, 2020). The negative correlation between responses was consistent with participants judging only one word in the dual-task condition, and guessing on the second word.

In this article, we investigated selective attention to words using the partially valid cueing paradigm (Posner, 1980). In partially valid cueing, a visual cue indicates where a target stimulus is most likely to appear. For valid cues, the target appears at the likely, cued location. For invalid cues, the target appears at the unlikely, uncued location. A cueing effect occurs when performance is better for valid cues than invalid cues.

Prior work has investigated the mechanisms underlying cueing effects, and the extent to which uncued information is processed. For tasks involving detection of simple features, such as a coarse discrimination task using Gabor patches, cueing effects are consistent with a weighted parallel model (Johnson et al., 2020) where information from cued and uncued locations is processed in parallel, but with more weight for the cued location in decision making. Weighted parallel models have been investigated extensively in the cueing literature (for further discussion, see Kinchla et al., 1995; Shimozaki et al., 2003).

Although cueing effects for simple features are consistent with a weighted parallel model, it is unclear whether cueing effects for more complex stimuli, such as words, are consistent with a parallel model or a serial model. McCann et al. (1992) conducted a series of experiments in which participants made lexical decisions on stimuli that appeared at cued or uncued locations. Half of the stimuli were words and half were pronounceable nonwords. The cue was 80% valid, meaning that the target appeared at the cued location on 80% of trials, and at the uncued location on 20% of trials. Participants were faster and more accurate at responding to the words when they appeared at the cued location compared to when they appeared at the uncued location.

McCann and colleagues' theoretical interest was to distinguish between early and late selection. They focused on two early selection models that might account for their results: one a serial model and the other a parallel model. The serial model assumes that attentional selection is allocated to the cued location; on invalid trials, when the target does not appear at that location, the selection mechanism must switch to the uncued location to process the target and make a response (their Type I model). This is a serial switching model in which attentional selection is allocated to different locations over time. For their parallel model, while information from the cued and uncued locations is processed in parallel, cued information is given greater processing in some way (their Type II model). Results were consistent with both models because both predict faster and more accurate responses for stimuli at the cued location than at the uncued location. One way to distinguish these two models is to limit attentional switching between the cued and uncued locations. When only one word is processed within a trial, results are consistent with an *all-or-none serial model*, which is a special case of serial switching.

The goal of the current study is to test whether cueing effects for words are consistent with the all-or-none serial model, or some kind of parallel model. Similar to McCann and colleagues, we used a semantic categorization task with partially valid cues. To distinguish the all-or-none serial model from parallel models, we used a postmask to minimize attentional switching as done by White et al. (2018). If the postmask prevents switching, the all-or-none serial model predicts chance performance for invalidly cued trials. In contrast, a parallel model in which attentional selection is allocated to both cued and uncued locations predicts above-chance performance for invalidly cued trials.

## Method

**Fig. 1:**
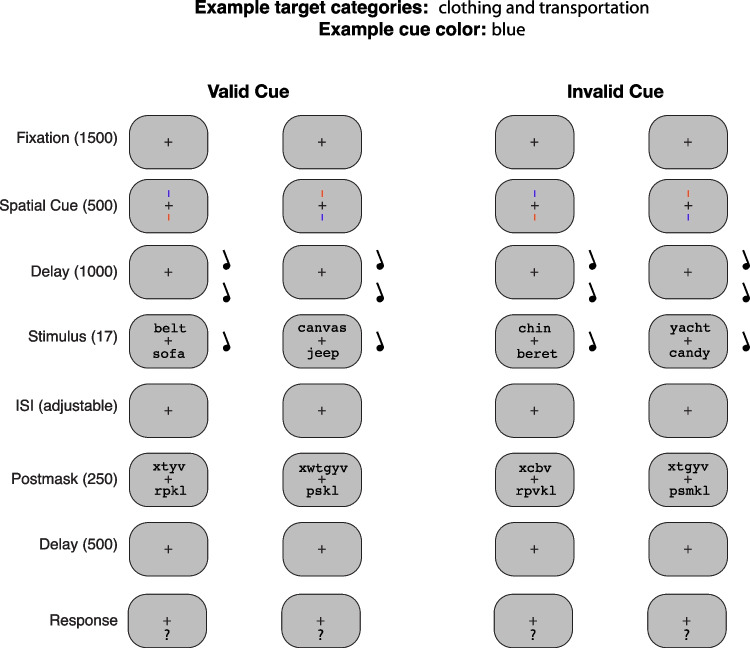
Schematic of four possible trial sequences shown in separate columns. Display durations are shown in milliseconds. In these example sequences, the cue color was blue. The task was to distinguish between two possible target categories (e.g., clothing and transportation) and ignore distractors from other categories (e.g., furniture). Two 500-Hz warning tones were played during the delay interval before the target, and a single 750-Hz one was played at the beginning of the target interval

An overview of the trials in this experiment is shown in Fig. 1. Each column illustrates an example trial. The two columns on the left show trials with valid cues and the two columns on the right show trials with invalid cues. The details of the sequence of the cue, the stimulus words, and the mask are described in the text below. The task was a forced-choice response between two semantic categories. For the example illustrated in the figure, the categories were clothing and transportation.

### Participants

There were 11 participants. All were paid and had normal or corrected-to-normal acuity. All gave informed consent in accord with the human subject Institutional Review Board at the University of Washington, in adherence with the Declaration of Helsinki.

To determine the appropriate sample size, we used data from a previously conducted partially valid cueing experiment (Johnson et al., 2020). In it, participants (*n* = 13) detected Gabor patches with methods similar to those in the current study. Based on the variability across participants observed in that study (sample standard deviation of the cueing effect = 7%), a power analysis was conducted to determine the sample size needed to detect a cueing effect the same size as found for the simultaneous display of that experiment (8%). Our calculations assumed an alpha error of 0.05 and a power of 95% (beta error of 0.05). The estimated minimum sample size was 10. To be conservative, we used 11 participants.

### Apparatus

Displays were presented on a linearized CRT monitor (Sony GDM-FW900) with resolution 1,024 × 640 pixels refreshing at 120 Hz. The monitor was viewed from 60 cm and the middle-gray background had a mean luminance of 56 cd/m^2^. Stimuli were created with MATLAB (MathWorks, Natick, MA, USA) and Psychophysics Toolbox (Brainard, 1997). Gaze position was monitored for all trials using an Eyelink 1000 (SR Research, Ottawa, Canada) and the Eyelink toolbox (Cornelissen et al., 2002). Trials without good fixation (e.g., broken fixations or blinks) were excluded from analysis. Such excluded trials were infrequent; across participants, they occurred on 1.9 ± 0.4% of trials for all conditions.

### Stimuli

Participants categorized words that appeared 1.5° above or below fixation. The linear distance between each word was 3°, which is larger than the distance at which visual crowding occurs, as expected from Bouma’s law (Bouma, 1970). The words were presented in nearly 100% contrast black Courier font (24 point) against a middle gray background. For one participant, the letter strings were presented in 25% contrast to increase the difficulty of the task. The word set used in this task was the same as that used in White et al. (2018). Words were drawn from 12 semantic categories: animals, anatomy, clothing, food, professions, transport, plants, buildings, music, household, environment, and materials. Each category consisted of 35 nouns ranging from four to six characters in length. The median lexical frequency of the words used was 6.4 per million, according to the Clearpond database (Marian et al., 2012). A postmask was used that consisted of random consonants. The number of characters in the mask matched the number of characters in each word presented within a trial.

### Task

To minimize any effect of response bias, we used a forced-choice discrimination task. Each participant was randomly assigned two target categories from which target words were drawn (e.g., clothing and transportation), and their target categories were the same throughout the duration of the experiment. Non-target distractor words were drawn from all of the ten remaining word categories. An example is shown in Fig. 1 in which the target categories were clothing and transportation, and the cue color was blue. In the leftmost column of Fig. 1, the target was “belt,” an example of clothing, and the distractor was “sofa,” an example of furniture. The correct response was the keypress corresponding to “clothing.” In the second column, the target was "jeep" and the distractor was "canvas," so the correct response was the keypress for "transportation."

### Procedure

The stimulus sequence for four example trials is shown in separate columns in Fig. 1. Each trial began with a fixation cross for 1,500 ms. A spatial cue was then shown for 500 ms. The cue consisted of two vertical lines, one above and one below fixation. Participants were assigned a cue color of red or blue. They were told that the cue color indicated which side of fixation (above or below) a target word was most likely to appear. To make the cue fully endogenous, the uncued side was assigned the other color. In the example shown in Fig. 1, which has a cue color of blue, participants were presented a blue vertical line on the cued side of fixation, and a red vertical line on the uncued side of fixation. The probability of the target appearing at the cued location was 0.8, and for the uncued location was 0.2.

Following the cue, there was a delay of 1,000 ms where only the fixation cross was visible. During the delay, two 500-Hz tones were played for 250 ms each, with a 250-ms delay between them. Beginning at the same time as the stimulus, a 750-Hz tone was played for 250 ms. The three regularly paced tones helped participants to anticipate when the target would occur, reducing temporal uncertainty (Johnson et al., 2020). Following the second delay, two words were presented, one above and one below fixation, for about 17 ms (two video frames). On valid trials, the target appeared at the cued location and the distractor appeared at the uncued location. On invalid trials, the distractor appeared at the cued location and the target appeared at the uncued location.

Following the stimulus, there was an inter-stimulus interval (ISI) of variable duration. During the practice sessions, the difficulty of the task was manipulated by the experimenter by varying the ISI for each participant such that performance in the valid cue condition was 70–80% correct. The average ISI across participants was 43.5 ms (range 17–104 ms). The ISI was followed by a postmask of random consonants for 250 ms. Following the mask, there was a delay of 500 ms where only the fixation cross was on the screen. Participants were then prompted to respond with a button press indicating which of the two target categories the target word was drawn from, and were given as much time as needed to do so.

For one participant, the procedure had slight differences to increase the difficulty of the task. The words and masks were presented at 25% contrast; the stimulus time and ISI were 50 ms. These differences in stimulus and procedure resulted in valid cue performance in the desired range of 70–80% correct.

Each experimental and control session consisted of 80 trials. This session length was chosen after pilot data showed that 80 trials limited participant fatigue from eye tracking. This design allowed participants to complete one to three sessions during an hour while taking breaks between sessions. All participants completed at least two training sessions, followed by ten experimental sessions and three control sessions. This design made for a total of 800 trials in the experimental condition and 240 trials in the control condition (described below).

### Control condition without effective masks

In addition to the experimental sessions, there were three control sessions in which the ISI before the mask was 1,000 ms for ten participants, and 600 ms for one early participant. The long ISI made the mask ineffective. The control sessions were conducted after participants completed all experimental sessions. The primary purpose of this control condition was to confirm that errors in the main condition were due to the postmask, and not to characteristics of the stimulus, such as difficulty discriminating the words. In addition, this control provided a comparison to the main condition to determine whether the results were specific to using an effective mask.

### Predictions

To better understand what models can be distinguished by this experiment, predictions were derived for three relevant models: the all-or-none serial model with optimal allocation, the all-or-none serial model with probability matching, and a representative fixed-capacity parallel model with optimal allocation. To make the prediction specific, we assumed parameters that yield 78% correct in the valid cue condition to set the difficulty of the judgment to match that observed in the experiment.

To begin, consider the all-or-none serial model with optimal allocation across the cued and uncued stimuli. This is the special case of the serial model that is our focus. In this model, only one stimulus is processed and it is always at the cued location. The cued stimulus is always processed because this yields the optimal allocation of processing for this model in this experiment. This model is robust to the detailed assumptions, such as the choice of evidence distribution. For further details see White et al. (2020). Performance for the valid cue condition is always the same as for a single-stimulus condition because the cued location is always fully processed on every trial. Performance for the invalid cue condition is always at chance because the uncued location is never processed. Thus, the specific predictions for this experiment are 78% correct for the valid cue condition (fixed to match the observed results), and 50% correct for the invalid cue condition (chance).

For the second model, consider the all-or-none serial model with probability matching. The idea of *probability matching* is from probabilistic choice tasks in which responses are often in proportion to the probability of a reward (reviewed in Shanks et al., 2002). Probability matching has been applied to a variety of situations including serial models of partially valid cueing (Jonides, 1983; van der Heijden, 1989). For this situation, the probability of processing a given location is in proportion to the cue validity. For example, given the cue is valid on 80% of the trials, the strategy is to process the cued location on 80% of the trials and process the uncued location on 20% of the trials. This strategy is not optimal for this task, but can be optimal for certain kinds of simple "foraging" tasks in which the trials are not independent and instead rewards accumulate at unvisited locations (Ellerby & Tunney, 2019). More generally, the probability-matching strategy illustrates a serial model that yields distinct predictions from the optimal allocation strategy.

Performance for each condition depends on a mixture of trials when the cued location was processed and trials when the uncued location was processed. The probability correct for the valid cue condition is given by:

$${p}_{correct}={c}_{valid} a+(1-{c}_{valid}) g,$$

where *c*_*valid*_ is the cue validity, *a* is the accuracy when the target is processed, and *g* is the accuracy when the target is not processed (guessing). For this experiment, *c*_*valid*_ = 0.8 and *g* = 0.5. With probability matching, the cued location is processed on *c*_*valid*_ of the trials. And for the valid condition, the target is always at the cued location that gives the first term of the equation. On the other 1-*c*_*valid*_ of the trials, the uncued location is processed. For the valid condition, the target is never at that location, so one can only guess which gives the second term of the equation. Given that we need the prediction given probability correct in the valid cue condition is 0.78, we can solve this equation for variable *a*, which yields *a* = 0.85.

Next consider the invalid cue condition in which the target is always at the uncued location. The probability correct for this condition is given by:

$${p}_{correct}=(1-{c}_{valid}) a+{c}_{valid}\; g,$$

which yields probability correct in the invalid condition of 0.57. Relative to the optimal allocation, probability matching raises the predicted performance for the invalid cue condition from 50% (chance) to 57% correct. This model illustrates how chance performance for the uncued stimulus depends on both the all-or-none serial model and an allocation rule that results in never processing the uncued stimulus.

For the third model, consider a representative parallel model that is often used to mimic the general serial model. For this model, the detailed assumptions do matter. Assume a fixed-capacity, parallel model that follows Shaw's (1980) sample size model. In the sample size model, the allocation of processing is varied by adjusting the relative number of samples for the cued versus uncued locations. Furthermore, assume the allocation of the samples is optimal in the sense that the overall performance is maximized. The optimal allocation of samples was calculated that maximized the average accuracy by trials over both valid and invalid conditions. Additional details of the decision rule followed the search model for two-target identification in Busey and Palmer (2008), and we assumed independent, equal-variance Gaussian distributions.

This model can be defined as follows: The judgment is based on a random variable *T* representing the net evidence for one target category (in positive values) or for the other target category (in negative values). The distractor is similarly represented by a random variable *D* that has a mean of zero. These evidence variables are assumed to be independent and normally distributed *N(m, s)* with their mean *m* and standard deviation *s*. Further assume that the evidence variables for the two categories are symmetric so that we can consider only the target stimuli that yield positive values. In this experiment, there is always one target and one distractor, so the probability correct *p*_*correct*_ is given by the equations:

$$\begin{array}{l}{p}_{correct}=P[ max(T, D) \ge max(-T, -D)],\\ T=N(d, {s}_{t}), and\\ D=N(0, {s}_{d}).\end{array}$$

In words, the main equation tests whether the evidence for the positive-valued category is greater than for the negative-valued category. These are correct responses because, given symmetry, we can restrict ourselves to stimuli from the positive-valued category. Thus far, the parameters are the discriminability of the target *d*, and the standard deviations of the target and distractor distributions *s*_*t*_ and *s*_*d*_.

In this partially valid cueing experiment, one of the stimulus locations is cued and the other is uncued. With the sample size model, the cue effect is due to shifting samples from the uncued location to the cued location. Specifically, let *w* be the fraction of the total number of samples devoted to the cued location and *1-w* be the fraction of samples devoted to the uncued location. Assuming the samples are independent, the variance of their sum is proportional to the number of samples. Further assume a unit value of the standard deviation when all samples are devoted to 1 stimulus. From this, the standard deviations are *s*_*cued*_ =$\sqrt{\frac{1}{w}}$ for the cued stimulus and *s*_*uncued*_ =$\sqrt{\frac{1}{1-w}}$ for the uncued stimulus. In summary, performance is a function of the discriminability parameter *d* and the weight *w*. This system of equations can be solved for probability correct as a function of *d* and *w* by either simulation or numerical integration.

To find the optimal allocation of samples, we can determine what value of *w* maximizes the overall probability correct, combining valid and invalid conditions on a by-trial basis. To do this, one can vary *d* and *w* to find the conditions that match the desired performance in the valid condition (78% correct observed in this experiment) and maximize the overall performance. Doing this calculation, the best overall performance is 74.5% correct with *w* = 0.891 and *d* = 0.818, which yields 78% correct in the valid cue condition and 60.6% correct in the invalid cue condition. Thus, this model predicts performance more than 10% above chance in the invalid cue condition.

## Results

**Fig. 2:**
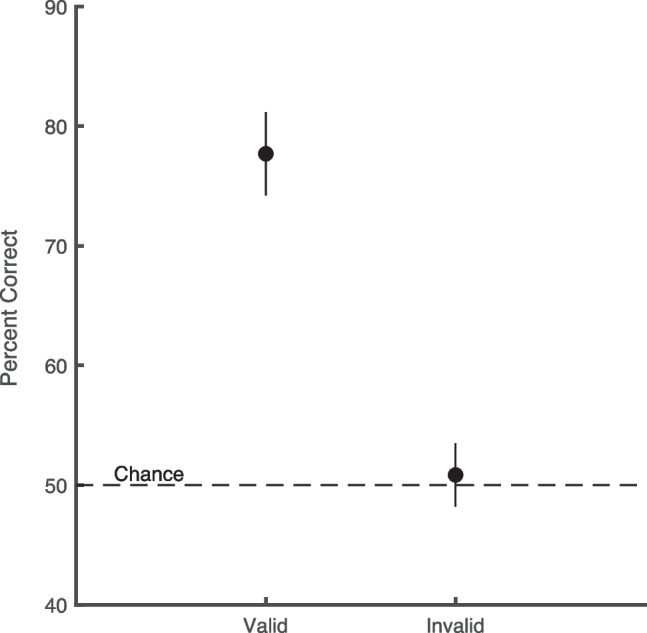
Percent correct for valid and invalid cues. Error bars represent 95% confidence intervals. Performance for the invalid condition was at chance

In Fig. 2, accuracy in terms of percent correct is shown for both valid and invalid cues. Chance performance for this categorization task was 50% correct. Performance in the valid condition was 77.8% and in the invalid condition is 50.8% for a cueing effect of 26.9% (95% CI: 21.0, 32.8), which was reliable (*t*(10) = 10.15, *p* < 0.001). Critically, performance in the invalid condition was at 50.8%, which is not reliably different from chance ((95% CI: 48.2, 53.5; *t*(10) = 0.71, *p* > 0.1). This result is consistent with the prediction of chance performance in the invalid condition for the all-or-none serial model. Performance on the invalid cue trials was also significantly lower than the prediction of 57% for the probability-matching model and the prediction of 60.6% correct for the optimal-allocation, fixed-capacity parallel model.

In the control condition without effective masks, performance in the valid cue condition was 96.1%, and in the invalid condition was 75.6%, for a cueing effect of 20.5% (95% CI: 10.1, 30.9), which was reliable (*t*(10) = 4.39, *p* < 0.001). Performance in the invalid condition without the mask was well above chance. Thus, performance in the main condition was limited by the postmask rather than by something such as the discriminability of the target word.

To examine these results more closely, we broke them down by word length. Our question was whether performance in the invalid cue condition was above chance for short words. For the sample of words displayed in the experiment, 29% of the words were four letters long, 39% were five letters long, and 32% were six letters long. Table 1 shows that even for the shortest words, performance in the invalid cue condition was 52.0% and not reliably different from chance (95% CI: 48.1, 56.0; *t*(10) = 1.14, *p* > 0.1). Thus, there is no sign that even short words are processed in the invalid cue condition.

**Table 1:** Cue effects as a function of target word length

		Word length	
	4	5	6
Valid Condition	79.3%	78.3%	75.5%
Invalid Condition	52.0%	51.2%	50.5%
Cue Effect	27.3%	27.1%	25.0%

To look further, we also subdivided the cueing conditions by the frequency of the target word in the language. While word frequency and word length are correlated (*r* = -0.175 for this set of words), there is plenty of room for an effect of frequency even if there is no effect of word length. Our specific question was whether performance in the invalid cue condition is above chance for high-frequency words. We used the word frequencies in the CLEARPOND database (Marian et al., 2012) to split the target words seen in the study into three groups with a roughly equal number of trials (~ 2,300 trials each). The high-frequency words had frequencies above 10.48 per million, low-frequency words were below 2.55 per million, and medium-frequency words were in between. This resulted in groups with mean word frequencies of 40.9, 5.4, and 1.1 per million. In Table 2, performance for the valid and invalid cue conditions is broken down by these three levels of word frequency. Even for high-frequency words, performance in the invalid cue condition was 51.8%, which is not reliably different from chance (95% CI: 47.8, 55.8; *t*(10) = 0.99, *p* > 0.1). Thus, there is no sign that even high-frequency words are processed in the invalid cue condition.

**Table 2:** Cue effects as a function of target word frequency

		Word frequency	
	High	Medium	Low
Valid Cue Condition	80.9%	78.0%	74.6%
Invalid Cue Condition	51.8%	50.1%	50.8%
Cue Effect	29.1%	27.9%	23.8%

In a final analysis, we examined practice effects to determine whether there was any improvement of the performance in the invalid cue condition. Such improvement would be expected if one learned to process words in parallel over the course of the experiment. Two complications must be discussed to interpret the practice effects in this experiment. First, there were a number of training sessions before any of the experimental sessions reported here. Thus, these effects reflect continuing practice rather than the initial practice in this task and using these stimuli. Second, to maintain a constant level of performance, we reduced the ISI during the course of the experiment if a participant showed signs of improvement in the valid cue condition. In fact, the ISI was reduced during the experiment for five of the 11 participants. Thus, performance changes in the valid cue condition say more about the success or failure of our adaptive procedure than about a simple practice effect.

There was no sign of a change in performance over the course of this experiment. Perhaps the most sensitive test was to split the ten sessions into two groups of five sessions. This amounts to comparing two blocks of 400 trials. For the valid cue condition, percent correct was 76.7 ± 3.1% in the first half and 78.7 ± 1.4% in the second half. This is an unreliable improvement of 2.0 ± 3.6% (95% CI: -6.1, 10.2; *t*(10) = 0.56, *p* = 0.59). For the invalid cue condition, percent correct was 51.5 ± 1.8% in the first half and 50.3 ± 1.9% in the second half for an unreliable decline of 1.2 ± 2.8% (95% CI: -7.5, 5.1; *t*(10) = 0.43, *p* = 0.68). Thus, our adaptive procedure to maintain constant difficulty was largely successful in the valid cue condition. More interestingly, there was no sign of improvement in the invalid cue condition. While there probably was a practice effect for the valid cue condition that was hidden by our adaptive procedure, there was no sign of improvement to above chance performance for the invalid cue condition.

## General discussion

We tested whether selective attention to words is consistent with an all-or-none serial model or with other models. Results showed a large cueing effect with chance performance for categorizing words at an uncued location. This is consistent with the all-or-none serial model, and rejects other models in which a reasonable amount of processing is given to uncued locations. Specifically, the results are not consistent with either a probability-matching version of the serial model or the fixed-capacity parallel model with optimal allocation. For a parallel model to result in chance performance at the uncued location, it must act like an all-or-none model and only process the cued word.

### Relation to prior studies of words and partially valid cueing

The cueing effect found in the current study is larger than that found in prior studies of partially valid cueing using words. One of the first studies to measure cueing effects for words was McCann et al. (1992). Invalid cue performance in that study was not at chance. In their Experiment 1, there were 10% errors in the valid condition and 15% errors in the invalid condition compared to a chance performance of 50% errors. There are a number of differences between the current study and McCann et al. (1992), so it is worth considering which of these differences might explain the chance performance for invalid cues found in the current study.

One difference between these studies is the use of accuracy rather than response time as the primary measure. But this is unlikely to be the critical difference because the control condition in the current study used accuracy with an ineffective mask, and did not find chance performance for the invalid condition. Thus, using accuracy as opposed to response time is not the critical difference.

Another difference between the studies is that McCann et al. (1992) presented a single target stimulus with no distractors during each trial (detection), rather than presenting both a target and a distractor (search). This is unlikely to be the critical difference because Cristescu and Nobre (2008) conducted a partially valid cueing experiment using unmasked words with an additional distractor similar to the current study. Despite this, Cristecu and Nobre did not find chance performance for the invalid condition. Similarly, our control condition with distractors and ineffective masks had accuracy well above chance for invalid cues. Thus, adding distractors and using a search task is not the critical difference.

We suggest that the critical difference among all of these studies is the use of postmasks. Postmasks were used in the current study to prevent attentional switching and to test the all-or-none serial model (see Shiu & Pashler, 1994; Smith, 2000, for reviews). An effective postmask interrupts processing such that information is available from the first stimulus to be processed, but not from the second (cf. Enns et al., 2001). Without a postmask, a serial model allows participants to switch attention from the cued location to the uncued location. This allows some processing of the word at the uncued location. It is only with an effective mask that this situation corresponds to the all-or-none special case of the serial model.

Might the postmask alone be the critical factor to obtaining chance performance? This seems unlikely because studies of partially valid cueing using postmasks and letters consistently find accuracy in the invalid condition to be well above chance (e.g., Shiu & Pashler, 1994). In summary, we argue that the chance performance for the invalid cue found in the current study, and not in prior cueing studies, is due to a combination of using word judgments and postmasks.

### Relation to prior studies of divided attention to words

The results observed in the current study are consistent with those of White et al., (2018, 2020), who found that in divided attention tasks requiring lexical or semantic judgments of words, participants could judge only one word and were at chance for a second word. In contrast, when naming the color of the word, participants could judge two words almost as well as one. In both judgments, postmasks followed the target stimulus; however, only the lexical and semantic judgments produced divided attention effects that were consistent with all-or-none serial processing. These results are consistent with the combination of the lexical or semantic judgment task and a postmask being effective to reveal the all-or-none serial model.

Our results are also consistent with prior work using the redundant target paradigm that found results consistent with the serial processing of words (Mullin & Egeth, 1989). In the redundant target paradigm (van der Heijden, 1975), one or two stimuli are presented that are either targets or distractors. The task is to respond "yes" when a target is present anywhere in the display and "no" otherwise. A self-terminating serial model predicts equal performance for the single- and two-target condition while typical parallel models predict a redundancy gain for the two-target condition. For judgments of simple features, such a redundancy gain has been found by many studies (e.g., Egeth et al., 1989; Krummenacher et al., 2002). However, for semantic judgments of words, Mullin and Egeth (1989) found no redundancy gain and interpreted their results as consistent with the serial processing of words (but see Shepherdson & Miller, 2014).

These issues have been addressed by two other lines of research that we can mention only briefly. The first are studies of speeded dual tasks using the PRP paradigm. Results consistent with a central bottleneck for words (and possibly serial processing) are described in McCann et al. (2000). For a more recent study that highlights individual differences in reading, see Ruthruff et al. (2008). The second line of research uses indirect measures such as priming and flanker tasks. Studies finding results consistent with the serial processing of words are reviewed by Lachter et al. (2004). A contrasting review consistent with parallel processing of words can be found in Snell and Grainger (2019).

### What processes are serial?

A natural question is what about the current experiment requires serial processing? In perception generally, one way that serial processing often arises is the need for eye movements to overcome the limits of peripheral vision, such as crowding (Yeatman & White, 2021). But in the current study, we are asking what might require serial processing within an eye fixation? We highlight three possible answers to this question. First, lexical and/or semantic tasks might require serial processing. This was part of Broadbent's (1958) early selection theory and is still viable today (Lachter et al., 2004). Second, perceiving relational information (e.g., spatial relations, or letter order) might require serial processing. This has been hinted at over the years (e.g., Logan, 1994; Moore et al., 2001; Palmer, 1994; Põder, 1999) and has been specifically proposed by Gilden et al. (2010). Third, perhaps the critical factor is not what requires serial processing, but instead what cannot be processed in parallel. By this view, serial processing is a fallback option for when parallel processing fails. This idea is part of the theory of guided search (Wolfe, 2007, 2021; Wolfe et al., 1989). In this theory, the parallel processing of stimulus information such as feature contrast (Nothdurft, 1993) can guide the selection of the target under many conditions. Under other conditions, however, parallel processing fails, such as with overwhelming stimulus heterogeneity (Rosenholtz, 2001). When it fails, one falls back on the serial processing of each stimulus. These three possibilities are not mutually exclusive and indeed all three might hold.

## Conclusion

We used partially valid cueing and a semantic categorization task to test whether multiple words can be semantically processed in parallel, or if processing can be allocated to only one stimulus at a time. In an experiment using masked words to prevent attentional switching, results showed both a large cueing effect and chance performance at the uncued location. Such chance performance is consistent with the all-or-none serial model in which only one word can be processed on a trial. This adds to previous evidence from dual-task and redundant-target paradigms that words are processed one at a time.
